# Space radiation impairs long‐term endothelial integrity and may promote atherosclerosis in ApoE‐deficient mice

**DOI:** 10.1002/ctm2.70774

**Published:** 2026-08-13

**Authors:** Yang Xu, Sabrina Jarrah, Shihong Zhang, Mary K. Khlgatian, Agnieszka Brojakowska, Yifei Du, Abrisham Eskandari, Valentina D'Escamard, Jason C. Kovacic, David A. Goukassian

**Affiliations:** ^1^ Cardiovascular Research Institute, Icahn School of Medicine at Mount Sinai New York New York USA; ^2^ Department of Cell Biology Yale School of Medicine New Haven Connecticut USA; ^3^ Victor Chang Cardiac Research Institute Darlinghurst Australia; ^4^ St. Vincent's Clinical School, University of NSW Darlinghurst Australia

1

Dear Editor,

Missions to the Moon and beyond are set to become an increasingly important focus of human space exploration. However, exposure to space radiation is an unavoidable hazard during space missions and may pose a significant risk.^1^ Studies of Apollo astronauts, the only humans with long‐term follow‐up data who have travelled outside Earth's protective magnetosphere, have suggested increased cardiovascular mortality.^2^ Although animal mechanistic studies support a causal role for radiation in cardiovascular injury,^3^ many studies to date have largely focused on acute or subacute effects.^4^, ^5^, ^6^ The long‐term cardiovascular consequences of space‐relevant radiation, particularly beyond one year after exposure and in the context of atherosclerosis, remain poorly defined.^3^ Moreover, most previous studies used single‐ion beam radiation rather than a mixed radiation field designed to mimic the complex composition of deep‐space radiation.^4^, ^5^

In this study, we addressed these gaps by investigating the long‐term effects of simulated space radiation on endothelial integrity and atherosclerotic remodelling. The development of NASA's galactic cosmic ray simulator for ground‐based testing enabled the use of simplified galactic cosmic ray simulation irradiation, referred to here as SimGCRSim, a mixed radiation field designed to better mimic deep‐space radiation exposure than traditional single‐ion analogues.^3^, ^6^, ^7^ We also included a gamma irradiation group, which provides an ‘Earth‐like radiation’ benchmark, allowing contextualization of the biological effects of space‐relevant SimGCRSim radiation. Atherosclerosis‐prone ApoE‐deficient mice were maintained on a normal chow diet prior to and following radiation and were sacrificed at 16 months of age for analyses (Figure 1A). For detailed methods, see Supporting Information.

**FIGURE 1:**
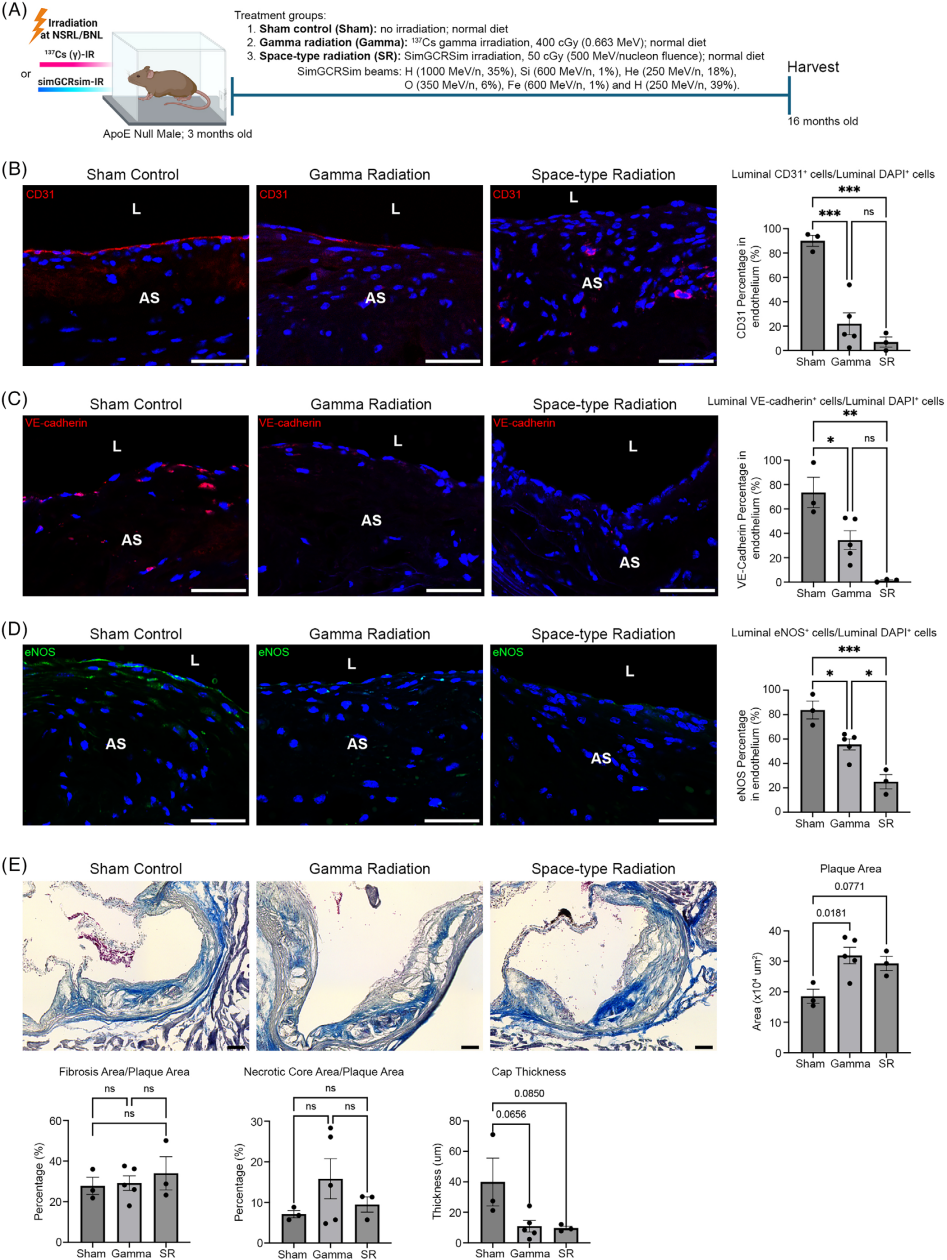
Simulated space radiation impairs endothelial integrity and may promote atherosclerosis in ApoE‐deficient mice. (A) Experimental design. IR, irradiation; SimGCRSim, simplified galactic cosmic ray simulation; NSRL, NASA Space Radiation Laboratory; BNL, Brookhaven National Laboratory. (B) Representative aortic root images of CD31 (red) and DAPI (blue) staining, with quantification. (C) Representative aortic root images of VE‐cadherin (red) and DAPI (blue) staining, with quantification. (D) Representative aortic root images of eNOS (green) and DAPI (blue) staining, with quantification. (E) Representative aortic root images stained with Masson's trichrome, with quantification of plaque area, fibrosis area, necrotic core, and fibrous cap thickness. Scale bars: 50 µm (B–D); 100 µm (E). *p ≤ .05; **p ≤ .01; ***p ≤ .001; ns, not significant. “L” indicates the lumen of the aortic root. “AS” indicates atherosclerotic plaque.

To evaluate endothelial integrity, we examined the expression of the endothelial markers CD31 and vascular endothelial (VE)‐cadherin in the luminal cell layer that was generally overlying aortic root atherosclerotic plaques. Immunofluorescence analysis revealed a significant reduction in CD31^+^ and VE‐cadherin^+^ endothelial coverage in irradiated mice compared with sham controls (Figure 1B,C). Both gamma radiation and SimGCRSim groups exhibited decreased endothelial coverage. This indicates significantly reduced arterial endothelial cell integrity more than one year after radiation exposure. We next examined expression of endothelial nitric oxide synthase (eNOS) in the endothelium of atherosclerotic plaques, a key regulator of vascular homeostasis and endothelial function. Consistent with the reduction in CD31^+^ and VE‐cadherin^+^ endothelial cell coverage, luminal eNOS expression was reduced in the gamma irradiated group compared with sham controls and further reduced in the SimGCRSim group (Figure 1D). These findings suggest that radiation exposure is associated with sustained impairment of endothelial nitric oxide signalling and endothelial integrity.

To assess the effects on atherosclerosis development, we quantified atherosclerotic plaque burden within the aortic root. Histological analysis demonstrated that mice exposed to gamma radiation developed significantly larger atherosclerotic plaques compared with sham controls (Figures 1E and 2B). Mice exposed to SimGCRSim showed a similar increase in plaque area, reaching borderline statistical significance (Figure 1E). These results suggest that both SimGCRSim at 50 cGy and gamma irradiation at 400 cGy likely promote atherosclerotic lesion formation, although the magnitude of the effect may depend on radiation type and dose. We further evaluated plaque composition to determine whether radiation alters plaque stability. Despite differences in plaque size, we did not observe significant changes in the proportion of fibrotic area or necrotic core area among the three groups. However, a trend toward reduced fibrous cap thickness was observed in both irradiated groups, which may suggest plaque destabilisation (Figure 1E). Additionally, plaque calcification was assessed using Von Kossa staining. Although not statistically significant, the SimGCRSim group exhibited approximately a two‐fold increase in plaque calcification compared with the sham control group (Figure 2A). Lipid content was evaluated using Oil Red O staining and no significant differences were observed (Figure 2B).

**FIGURE 2:**
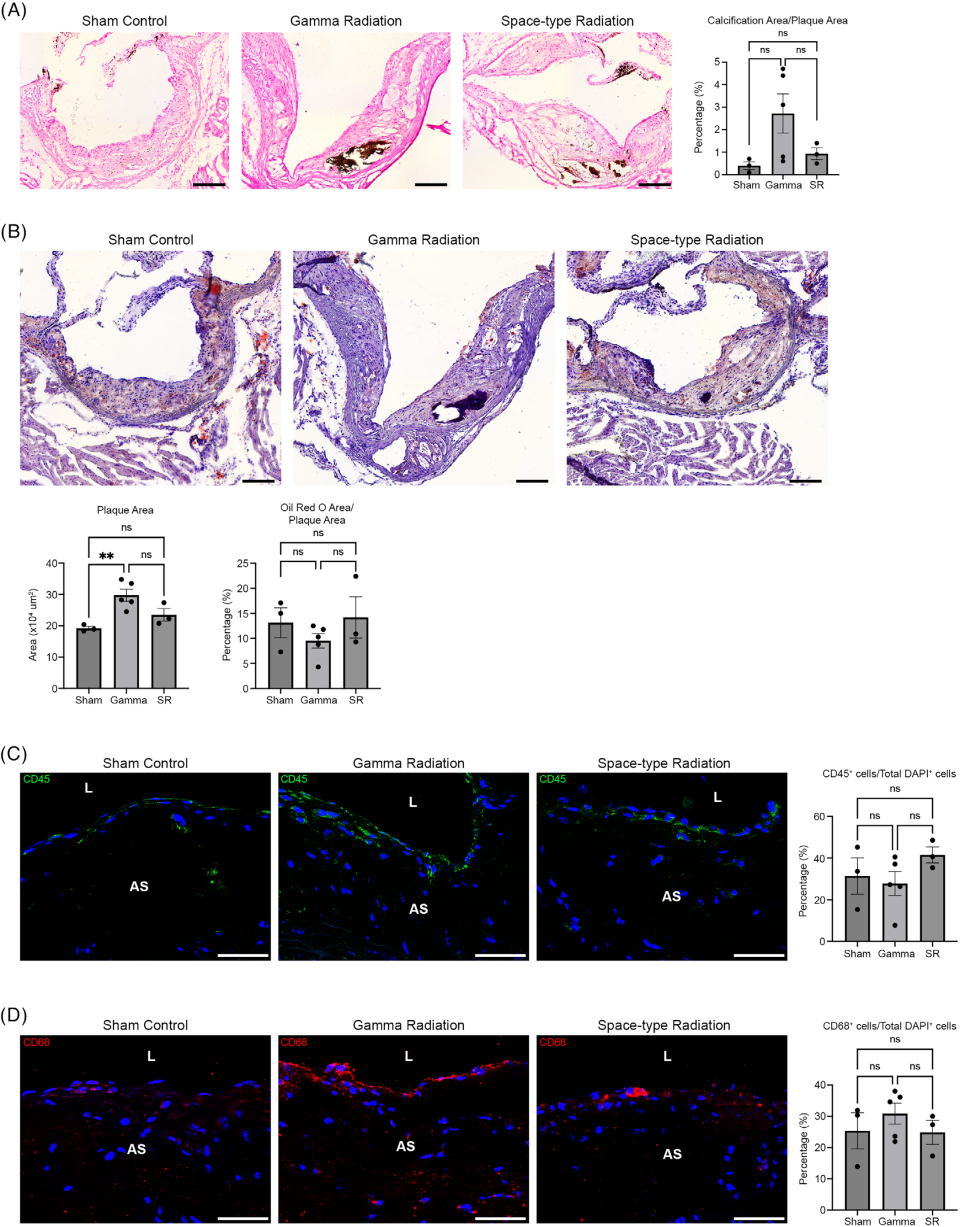
Plaque calcification, lipid content, and inflammatory cell infiltration following radiation exposure. (A) Representative images of aortic root sections using Von Kossa stain (black indicates calcification), with quantification of calcification. (B) Representative Oil Red O staining of aortic root sections and quantification of lipid content (red) in plaques. (C) Representative aortic root images of CD45 (red) and DAPI (blue) staining, with quantification. (D) Representative aortic root images of CD68 (green) and DAPI (blue) staining, with quantification. Scale bars: 200 µm (A, B); 50 µm (C, D). **p ≤ .01; ns, not significant. “L” indicates the lumen of the aortic root. “AS” indicates atherosclerotic plaque.

To determine whether inflammatory cell infiltration contributed to the observed changes, we examined leukocyte and macrophage content within atherosclerotic plaques. Immunofluorescence staining for CD45 and CD68 revealed comparable levels of leukocyte and macrophage accumulation, respectively, across all experimental groups (Figure 2C,D), indicating that radiation exposure did not substantially alter inflammatory cell infiltration within plaques in this long‐term model.

Most prior studies investigating radiation‐induced vascular injury have used single‐ion iron‐ion irradiation and focused on early or intermediate time points following irradiation, typically ranging from days to several months after exposure (reviewed here^4^, ^6^, ^8^). To our knowledge, this is the first study to use NASA's SimGCRSim to investigate long‐term atherosclerotic plaque biology. We demonstrate that mixed simulated space‐type radiation persistently impairs the endothelial cell lining of atherosclerotic plaques and may promote atherosclerosis development long after radiation exposure. The reductions in endothelial coverage (luminal CD31^+^ and VE‐cadherin^+^ cells) and eNOS expression in the endothelial layer indicate sustained impairment of endothelial integrity and nitric oxide signalling. Importantly, these vascular alterations were detected more than one year after irradiation, suggesting that space‐relevant radiation exposure produces lasting vascular injury. These findings align with previous research showing that single‐ion (iron‐ion) radiation accelerates atherosclerosis over a relatively short‐term.^5^

While we did not extensively explore biologic mechanisms in this pilot study, persistent eNOS reduction may indicate impaired nitric oxide bioavailability, a hallmark of chronic endothelial dysfunction that disrupts vascular tone, inflammatory regulation, and vascular homeostasis. Previous studies have linked age‐related nitric oxide loss to oxidative stress, mitochondrial dysfunction and vascular stiffening.^9^, ^10^ Space radiation may therefore activate vascular‐ageing pathways by inducing mitochondrial reactive oxygen species, thereby impairing ATP production, redox signalling and endothelial function. Further studies are warranted to define these mechanisms and determine how space radiation contributes to the persistent loss of endothelial integrity.

In conclusion, simulated space‐type radiation exposure impairs long‐term endothelial integrity and nitric oxide signalling and may promote atherosclerotic lesion development in ApoE‐deficient mice. These findings provide initial evidence supporting a role for space radiation in enhancing long‐term cardiovascular risk during deep‐space missions. This likely has major implications for future space exploration. As we embark on missions to return humans to the Moon and beyond, additional studies are urgently needed to fully define the long‐term effects of space radiation exposure on vascular dysfunction and atherosclerosis.

## AUTHOR CONTRIBUTIONS

Yang Xu conceived, performed and analysed experiments, prepared figures and co‐wrote the manuscript. Sabrina Jarrah, Shihong Zhang, Mary K. Khlgatian, Agnieszka Brojakowska, Yifei Du, Abrisham Eskandari and Valentina D'Escamard helped prepare the animals, cryosectioning and staining. Jason C. Kovacic and David A. Goukassian conceived the study, supervised experiments and data analysis, and co‐wrote the manuscript. All authors edited the manuscript.

## CONFLICT OF INTEREST STATEMENT

The authors declare no conflict of interest.

## ETHICS STATEMENT

All animal procedures were performed in accordance with the guidelines of the Guide for the Care and Use of Laboratory Animals for the National Institutes of Health and approved by the Animal Care and Use Committees at Brookhaven National Laboratory (Upton, NY, USA) (BNL IACUC Protocol #502) and the Icahn School of Medicine at Mount Sinai (New York, NY, USA) (ISMMS IACUC Protocol #2019‐0017).

## Supporting information



Supporting Information

## Data Availability

The data underlying this article will be shared upon reasonable request to the corresponding authors.
