# Supplementary material for: Space radiation impairs long‐term endothelial integrity and may promote atherosclerosis in ApoE‐deficient mice
Source: Clin Transl Med. 2026 Aug 13;16(8):e70774. doi: 10.1002/ctm2.70774 (PMC13470029; doi:10.1002/ctm2.70774)
Supplement: Supplementary file 1 [file CTM2-16-e70774-s001.docx]

**SUPPLEMENTAL MATERIALS**

**Space Radiation Impairs Long-Term Endothelial Integrity and May Promote Atherosclerosis in ApoE-Deficient Mice**

**Methods**

Animals and treatments. Three-month-old male ApoE^-/-^ mice were exposed to either sham irradiation (no radiation, n=3), gamma irradiation (^137^Cs, 400 cGy, 0.663 MeV, n=5), or simplified galactic cosmic ray simulation irradiation (SimGCRSim, 50 cGy, 500 MeV/nucleon fluence, n=3) at the NASA Space Radiation Laboratory (NSRL). NASA’s standard SimGCRSim protocol uses 6 beams and 5 ions (protons at 2 different energies) and runs for approximately 20 minutes. The ions are delivered in the following order as ion (energy, fraction): H (1000 MeV/n, 35%), Si (600 MeV/n, 1%), He (250 MeV/n, 18%), O (350 MeV/n, 6%), Fe (600 MeV/n, 1%) and H (250 MeV/n, 39%). Of importance, the radiation modalities and doses utilized in this study of 400 cGy of gamma radiation and 50 cGy of simulated galactic cosmic rays (simGCRsim) represent physically and biologically distinct exposures and were not chosen to facilitate a direct, equivalent dose comparison. The 50 cGy SimGCRSim exposure was selected to model a highly relevant, cumulative dose scenario realistic for long-duration deep-space missions, capturing the complex, high-LET (linear energy transfer) track structure characteristic of the space environment. In contrast, the 400 cGy gamma exposure serves as a high-dose, low-LET reference benchmark intended to elicit a robust, well-characterized biological response. Because these exposures differ significantly in dose, dose rate, and radiation quality, this study is not designed to establish relative biological effectiveness (RBE) or directly equate the two modalities. Instead, our focus is on characterizing the unique phenomenological outcomes of a space-relevant GCR simulation compared to standard terrestrial radiation baseline, ensuring a more cautious and accurate interpretation for spaceflight risk modeling.

All animals were euthanized by cardiac puncture and exsanguination under isoflurane inhalation anesthesia (NDC 10019-360-40, Baxter Healthcare). After tissue harvesting, aortic roots were incubated in 20% sucrose in phosphate-buffered saline (PBS) at 4 °C overnight before embedding in OCT compound (62550-01; Electron Microscopy Sciences) and stored at -80°C. The aortic roots were then cryosectioned at a thickness of 10 μm using a cryostat in the same orientation (CM3050S; Leica) onto precleaned glass slides (12-550-19; Fisher Scientific) and stored at -80°C.

Immunofluorescence analysis. Frozen sections were thawed at room temperature and rinsed in PBS to remove OCT and then fixed with 4% paraformaldehyde for 10 minutes. For immunofluorescence staining, samples were blocked in 5% bovine serum albumin for 60 minutes and then incubated overnight at 4°C with primary antibodies in antibody diluent (S080983-2, DAKO, Carpinteria, CA, USA) at the specified concentrations (see Supplemental Table 1). Sections were then washed three times in PBS and incubated for 1 hour at room temperature with Invitrogen Alexa Fluor™ 488 and/or Alexa Fluor™ 546 secondary antibodies. Finally, slides were washed three times with PBS and mounted with mounting medium containing DAPI (H-1200, Vector Laboratories, Newark, CA, USA). Images were acquired using a confocal microscope (LSM780, Zeiss). Data were averaged from at least two separate sections per animal. ZEN software from Zeiss was used to analyze the image. Luminal CD31, VE-cadherin and eNOS were quantified along the mono-cell layer overlying atherosclerotic plaques in the aortic root as previously described^1^. All quantifications were performed in a blinded manner.

Histological analysis. Aortic root sections were stained with Oil Red O (ab150678, Abcam), Masson’s trichrome (HT15, MilliporeSigma), and Von Kossa stain (ab150687, Abcam) according to the manufacturer’s instructions. Images were acquired using a Leica DMi8 microscope. Fiji software (version 2.0.0-rc-68/1.52w for Mac) was used for image analyses. All quantifications were performed in a blinded manner.

Statistics. No outliers or data points were excluded from this study. For all statistical analyses, each animal was used as the biological unit of analysis. For each animal, at least two separate aortic root sections were analyzed and averaged, and each dot in the quantification graph represents one animal. We first tested the data sets for normality and equal variance. For normal distributed data with equal variance, one-way ANOVA with post hoc Tukey’s multiple comparison test was used. Otherwise, Kruskal-Wallis test with post hoc Dunn’s multiple comparison test was used. Statistical analyses were performed using Prism 10, and a 2-sided P value of less than 0.05 was considered significant. All data are presented as mean ± SEM. Additional details regarding the statistical analyses are provided in Supplemental Table 2.

**Limitations**

Several limitations of this study should be considered. First, this was a preliminary investigation with limited sample size. This was largely because there is currently only one official ground-based galactic cosmic ray simulator facility in the United States located at NASA Space Radiation Laboratory/Brookhaven National Laboratory and the need to allocate animals across our multiple concurrent projects^2,3^. Second, although we observed trends toward increased plaque burden and lesion calcification following SimGCRSim exposure, larger studies will be necessary to determine the full extent of these effects. Particularly, although gamma irradiation produced a clearer increase in plaque area, the SimGCRSim-associated increase in plaque area reached only borderline statistical significance. Additional plaque-burden assessments, including en face aortic lesion analysis, serial aortic root sectioning, plasma lipid profiling, and body weight would need to be included in future studies when available. Third, while the current study showed reduced expression of mature endothelial markers and impaired endothelial integrity, no overt coagulation events or thrombi were detected at the time of harvest. The fate of endothelial-layer cells that lose mature endothelial marker expression remains unclear and warrants further investigation.

**Sex as a Biological Variable**

Male ApoE-deficient mice were used in this study. Male mice were selected because they develop robust and reproducible atherosclerotic lesions in aging ApoE^-/-^ models while allowing long-term survival required for extended observation periods. The use of a single sex in this pilot study also reduced biological variability and allowed clearer interpretation of radiation-induced vascular effects in the context of long-term aging. Although only male mice were examined, the mechanisms of radiation-induced endothelial dysfunction - such as impaired endothelial integrity and reduced nitric oxide signaling - are fundamental vascular processes that are expected to be relevant in both sexes. Nevertheless, potential sex-specific responses to space-type radiation cannot be excluded, and future studies including both male and female mice will be necessary to determine whether sex differences influence the cardiovascular effects of space-type radiation exposure.

**Supplemental Table 1. Antibody Details**

| **Antibody** | **Company** | **Catalog number** | **Concentration used** |
| --- | --- | --- | --- |
| CD31 | BD Biosciences | 550274 | 0.31 μg/mL |
| VE-cadherin | Novus | NBP1-43347 | 10 μg/mL |
| eNOS | Abcam | ab300071 | 5.49 μg/mL |
| CD45 | Abcam | ab10558 | 10 μg/mL |
| CD68 | Bio-Rad | MCA1957 | 10 μg/mL |

**Supplemental Table 2. Statistical Details**

| Figure | Mean ± SEM* | Statistical Method | Multiple Comparison Groups | Adjusted Multiple Comparison *P* Value |
| --- | --- | --- | --- | --- |
| 1B | 89.93% ± 4.49% | Ordinary one-way ANOVA | Sham vs. Gamma | 0.0007 |
|  | 21.92% ± 8.99% |  | Sham vs. SR | 0.0004 |
|  | 6.97% ± 4.16% |  | Gamma vs. SR | 0.4095 |
|  |  |  |  |  |
| 1C | 73.57% ± 12.44% | Ordinary one-way ANOVA | Sham vs. Gamma | 0.0267 |
|  | 34.48% ± 7.71% |  | Sham vs. SR | 0.0016 |
|  | 1.37% ± 0.70% |  | Gamma vs. SR | 0.0556 |
|  |  |  |  |  |
| 1D | 83.80% ± 7.31% | Ordinary one-way ANOVA | Sham vs. Gamma | 0.0171 |
|  | 55.52% ± 4.48% |  | Sham vs. SR | 0.0004 |
|  | 24.93% ± 5.84% |  | Gamma vs. SR | 0.0114 |
|  |  |  |  |  |
| 1E Plaque Area | 185353 ± 23032 | Ordinary one-way ANOVA | Sham vs. Gamma | 0.0181 |
|  | 319145 ± 27104 |  | Sham vs. SR | 0.0771 |
|  | 293057 ± 23523 |  | Gamma vs. SR | 0.7735 |
|  |  |  |  |  |
| 1E  Fibrosis/  Plaque | 27.83% ± 4.22% | Ordinary one-way ANOVA | Sham vs. Gamma | 0.9763 |
|  | 29.32% ± 3.54% |  | Sham vs. SR | 0.7227 |
|  | 34.07% ± 8.17% |  | Gamma vs. SR | 0.7881 |
|  |  |  |  |  |
| 1E  Necrosis/  Plaque | 7.10% ± 0.91% | Kruskal-Wallis | Sham vs. Gamma | >0.9999 |
|  | 15.82% ± 4.94% |  | Sham vs. SR | >0.9999 |
|  | 9.47% ± 1.88% |  | Gamma vs. SR | >0.9999 |
|  |  |  |  |  |
| 1E Cap Thickness | 39.87 ± 15.67 | Ordinary one-way ANOVA | Sham vs. Gamma | 0.0656 |
|  | 10.88 ± 3.75 |  | Sham vs. SR | 0.0850 |
|  | 9.60 ± 1.27 |  | Gamma vs. SR | 0.9924 |
|  |  |  |  |  |
| 2A | 0.40% ± 0.17% | Ordinary one-way ANOVA | Sham vs. Gamma | 0.1182 |
|  | 2.72% ± 0.87% |  | Sham vs. SR | 0.8884 |
|  | 0.93% ± 0.26% |  | Gamma vs. SR | 0.2464 |
|  |  |  |  |  |
| 2B  Plaque Area | 191987 ± 6138 | Ordinary one-way ANOVA | Sham vs. Gamma | 0.0087 |
|  | 297701 ± 19200 |  | Sham vs. SR | 0.3494 |
|  | 234754 ± 20083 |  | Gamma vs. SR | 0.0930 |
|  |  |  |  |  |
| 2B  Oil Red/ Plaque | 13.13% ± 2.98% | Ordinary one-way ANOVA | Sham vs. Gamma | 0.6034 |
|  | 9.54% ± 1.46% |  | Sham vs. SR | 0.9628 |
|  | 14.20% ± 4.13% |  | Gamma vs. SR | 0.4424 |
|  |  |  |  |  |
| 2C | 31.37% ± 8.70% | Ordinary one-way ANOVA | Sham vs. Gamma | 0.9149 |
|  | 27.76% ± 5.72% |  | Sham vs. SR | 0.5886 |
|  | 41.50% ± 3.81% |  | Gamma vs. SR | 0.3246 |
|  |  |  |  |  |
| 2D | 25.37% ± 5.75% | Ordinary one-way ANOVA | Sham vs. Gamma | 0.6346 |
|  | 30.84% ± 3.36% |  | Sham vs. SR | 0.9968 |
|  | 24.87% ± 3.86% |  | Gamma vs. SR | 0.5852 |
| *Mean ± SEM values are presented from top to bottom for each of the figure as sham, gamma, and space radiation (SR) groups, respectively. | | | | |

**Reference of Supplemental Materials**

1. Xu Y, Korayem A, Cruz-Solbes AS, Chandel N, Sakata T, Mazurek R, Mavropoulos SA, Kariya T, Aikawa T, Yamada KP, D'Escamard V, V'Gangula B, Baker AH, Ma L, Björkegren JLM, Fuster V, Boehm M, Fish KM, Tadros R, Ishikawa K, Kovacic JC. Inhibition of endothelial-to-mesenchymal transition in a large animal preclinical arteriovenous fistula model leads to improved remodelling and reduced stenosis. Cardiovasc Res. 2024 Nov 25;120(14):1768-1779.

2. Zakharyan R, Hakobyan S, Brojakowska A, Davitavyan S, Stepanyan A, Sirunyan T, Khachatryan G, Khlgatian MK, Bisserier M, Zhang S, Sahoo S, Hadri L, Garikipati VNS, Arakelyan A, Goukassian DA. Long-lasting sex-specific alteration in left ventricular cardiac transcriptome following gamma and simGCRsim radiation. Sci Rep. 2025 Feb 18;15(1):5963.

3. Brojakowska A, Jackson CJ, Bisserier M, Khlgatian MK, Grano C, Blattnig SR, Zhang S, Fish KM, Chepurko V, Chepurko E, Gillespie V, Dai Y, Lee B, Garikipati VNS, Hadri L, Kishore R, Goukassian DA. Lifetime Evaluation of Left Ventricular Structure and Function in Male C57BL/6J Mice after Gamma and Space-Type Radiation Exposure. Int J Mol Sci. 2023 Mar 13;24(6):5451.
